# Spondylodiscitis in Geriatric Patients: What Are the Issues?

**DOI:** 10.1177/21925682221121300

**Published:** 2023-04-21

**Authors:** Christian Herren, Nicolas Heinz von der Hoeh, Stefan Zwingenberger, Daniel Sauer, Norma Jung, Philipp Pieroh, Steffen Drange, Matthias Pumberger, Max J. Scheyerer, and Spine Section of the German Society for Orthopaedics and Trauma

**Affiliations:** 1 Department of Orthopaedics, Trauma and Reconstructive Surgery, 39058 RWTH Aachen University, Germany; 2 Department of Orthopedic, Trauma and Plastic Surgery, 9180 University Hospital Leipzig, Germany; 3 University Center of Orthopedic, Trauma and Plastic Surgery, 39063 University Hospital Carl Gustav Carus at TU Dresden, Germany; 4 Spinecenter, 40571 Schön Klinik Munich Harlaching University, Deutschland; 5 Department I of Internal Medicine, Medical Faculty, University Hospital of Cologne, University of Cologne, Germany; 6 Department of Orthopaedic Surgery, Klinikum Magdeburg gGmbH, Germany; 7 Spine Department, Center for Musculoskeletal Surgery, 14903 Charité University Medicine BerlinUniversity, Germany; 8 Department of Orthopedic and Trauma Surgery, Medical Faculty, University of Cologne, Germany

**Keywords:** spondylodiscitis, geriatric patients, conservative, operative, comorbidities, resistance-based antibiotics

## Abstract

**Study Design:**

Review article.

**Objectives:**

A review of literature on the treatment of pyogenic spondylodiscitis in geriatric patients was performed with the aim to give an overview about these special patients and a recommendation on necessary diagnostics as well as conservative and operative treatment options.

**Methods:**

A systematic computerized literature search was done by the spondylodiscitis working group of the German Society for Orthopedics and Trauma Surgery.

**Results:**

Spondylodiscitis has an increasing incidence by age with a peak at 75 years or older. The 1-year mortality without an appropriate treatment is with 15 to 20% extremely high. Pathogen detection is the essential diagnostic step and the basis for a sufficient antibiotic treatment. Geriatric patients have initially less elevated inflammatory parameters. Compared to younger patients. They have a longer length of hospital stay and take longer for CRP normalization. Even the outcome between conservative and operative treatment is comparable after one year. Patients with spinal instability, immobilizing pain, epidural abscess, and newly emerged neurological deficits should be considered for operative treatment.

**Conclusions:**

The treatment of geriatric patients with pyogenic spondylodiscitis must take into account that these patients usually have multiple comorbidities. The main goals are resistance-based antibiotics and the shortest possible time of immobilization of the patient.

Spondylodiscitis Study Group, Spine Section of the German Society of Orthopaedic and Trauma Surgeons

## Introduction

With increasing age in Western countries, the likelihood of secondary diseases such as neoplasia, metabolic diseases and susceptibility to infections increases. A significant influence seems to be the decline in the efficiency of the own immune system, the so-called immunosenescence.^1,2^ With regard to all age groups, pyogenic spondylodiscitis occurs most frequently in 70-79 years old patients. Moreover, the incidence of spondylodiscitis is estimated .4 – 2.4 per 100 000 per year.^3^ Several studies point to an increasing incidence due to an aging population with up to 11.3/100 000.^4,5^ Besides reports on a bimodal age-distribution 40-50 years ago,^6-9^ today there seems to be a rising evidence for a peak mainly in the population aged 75 years and older.^5^ Without an appropriate treatment, the overall mortality rate is reported in literature to be as high as 15-20%. However, the reason for this is the often long period between the onset of the disease and the final diagnosis with initiation of the adequate therapy.^10^ Unspecific symptoms and the absence of clinical presentations such as fever at diagnosis and initiation of therapy are among the reasons for the delay. With the growing population of multimorbid and immunocompromised patients in particular, spondylodiscitis should be included in differential diagnostic considerations at an early stage, even in the presence of nonspecific symptoms. For this reason, we included key points on the diagnosis and treatment of pyogenic spondylodiscitis in this review as part of a comprehensive systematic review also considering the current guidelines of the Infectious Disease Society of America (IDSA) and the German guidelines.

## Material and Methods

### Study Design

We conducted a comprehensive systematic review of the literature according to the Preferred Reporting Items for Systematic Reviews and Meta-analyses (PRISMA) checklist and algorithm.^11^

### Study Characteristics

Investigations between 2000 and 2020 were included. For analyses, prospective and retrospective observational studies that dealt with spondylodiscitis in geriatric patients were considered. Furthermore, only articles in German or English language were included.

### Information Sources

The authors performed an initial search of PubMed, Google scholar and Cochrane databases for investigations for possible inclusion in the review.

### Search

The keywords used in the research were ((“elderly”) OR (“older age”) OR (“geriatric”) OR (“aged”)) AND ((“spondylodiscitis”) OR (“discitis”) OR (“Spinal infection”) OR (“Spinal Osteomyelitis”) OR (“Pyogenic spinal infection”)

### Study Selection

**Figure 1.**
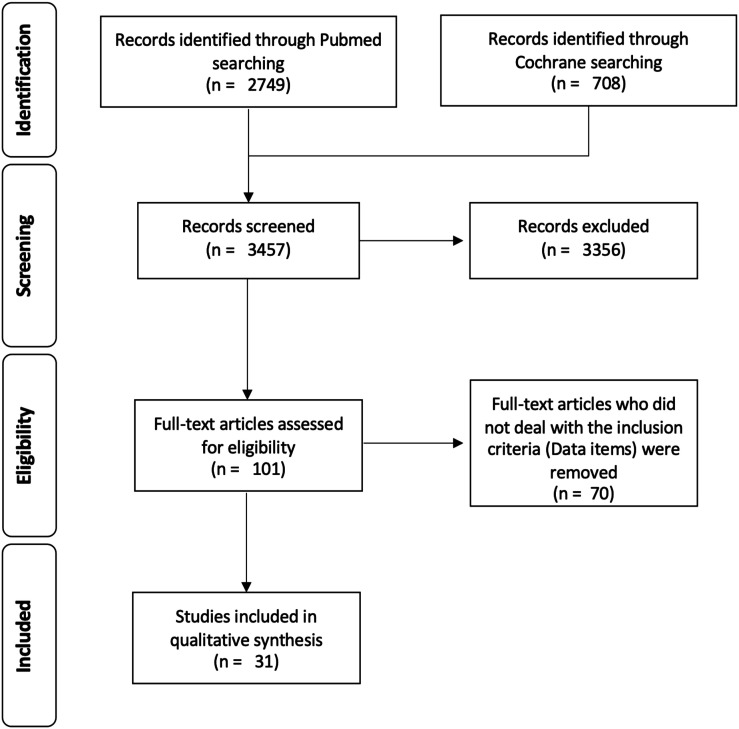
Prisma Flow chart.

The authors limited the research to observational studies, while systematic reviews, meta-analyses, case series and case reports were excluded. Titles and abstracts were reviewed. Duplicates were removed and full texts were checked for suitability. In cases where a decision could not be taken based on information from the title and abstract, the full text was evaluated. The final decision was made based on the analysis of the full text. The study selection process was carried out independently by three authors (NHvdH, CH, MJS) (Figure 1).

### Data Items

The main prerequisite was that included studies dealt with spondylodiscitis in elderly >65 years. The authors performed an initial search of the above-mentioned databases for investigations for possible inclusion in the review. First, title and abstracts were screened. In cases where a decision could not be taken due to the information from the title and abstract the full text was evaluated. In the end, the final decision was made based on the analysis of the full text. Studies were selected according to the following inclusion criteria: (a) Incidence, (b) diagnostic workup, (c) non-surgical therapy, (d) surgical therapy.

### Synthesis of Results

We extracted data concerning study characteristics including author’s name, title, year of publication and journal published. Outcome parameters were analysed according to the abovementioned inclusion criteria. For all included studies, we used the Oxford Centre for Evidence-Based Medicine (2011) guidelines for defining the level of evidence. The strength of recommendation was defined using the GRADE approach (Grading of Recommendations Assessment, Development and Evaluation). Regarding the risk of bias every included investigation was graded with respect to the Newcastle Ottawa scale (Table 1).

**Table 1.** Study characteristics of the included Investigations.

#	Puiblicationdate	Authors	Main findings	No. of patients	Type of study	EBM-Level	GRADE	NewcastleOttawa Scale
1	2009	Kapsalaki E. et al.	Spontaneous spondylodiscitis should be considered in every patient with back pain accompaniedby fever and laboratory markers of inflammation.	8	review	IV	low	n/a
			The major predisposing risk factor seems to be uncontrolled diabetes.					
2	2008	Yang S.C. et al.	Perkutaneous endoscopy is superior to CT-guided biopsy for identifyen pathogen	52	therapeutic study	III	moderate	***
3	2009	Deininger, M.H. et al.	minimally invasive percutaneous fixation is a feasible and effective technique to achieve immediate pain release,avoid long-term immobilization and overcome the disadvantages of a dorsoventral procedure.	12	prospective cohort study	III	moderate	***
4	2004	Mann, S. et al.	Surgical treatment is the modality of choice in patients with acute spinal osteomyelitis. The decision whether an anterior or posterior approach should be used must be made on an individual basis.	24	prospective cohort study	III	moderate	**
5	2006	Frangen, T.M. et al.	Initial surgical treatment included posterior internal fixation only. Abscesses can be drained percutaneously.Ventral debridement and stabilization is only recommended if insufficient stability can be obtained by dorsal fixation alone, as shown by the persistence of infection or pain.	78	retrospective study	IV	low	*
6	2007	Chaudhary, S.B. et al.	Early detection and aggressive treatment are paramount inmanaging postprocedural spinal infections and limiting their long-term sequelae.	n/a	review	IV	low	n/a
7	2008	Hutchinson, C. et al.	The mean time from symptom onset to diagnosis was 34 days.Most patients presented with back pain and elevated CRP.Differentiation between discitis and other spinal infections does not appear to be important	41	retrospective study	IV	low	*
8	2007	Butler, J.S. et al.	In the majority of cases, conservative management of pyogenic spinal infectionwith antibiotic therapy and spinal bracing is very successful.	48	retrospective study	IV	low	*
9	2009	Robinson, Y. et al.	This study reveals healing and improved function after expandable titanium cage implantation in all patients.	25	prospective study	III	moderate	***
10	2007	Suess, O. et al.	Same-stage instrumentation allows early postoperative mobilization of the patient, which is advantageous,especially for an increasingly elderly population and in patients with comorbidities.	24	retrospective study	IV	low	**
11	2009	Maus, U. et al.	PCT is not useful as diagnostic tool or monitoring parameter for spondylodiscitis.	35	prospective study	III	moderate	***
12	2008	Robinson, Y. et al.	Prerequisites fur the usefulness of titanium cages are a radical debridement, correction of deformity, and additional bony fusion by bone grafting.	22	prospective study	III	moderate	***
13	2010	Uchida, K. et al.	In case of neurological impairment an urgent MRI staging regimen should be started.	37	retrospective study	IV	low	*
14	2015	Dreimann, M. et al.	Within al follow-up time of 19 months, the the modified posterior transversectomy with 360-degree decompression and anterior wall reconstruction with titanium cages in combination with posterior instrumentationfor sagittal alignment correction is a reliable, effective, and safe treatment option.	10	retrospective study	V	Very low	n/a
15		Yoon, Y.K. et al.	Differentiation between TBC and Pyogenic Spondy, risk index from WBc, PCT and CrP	117	retrospective study	IV	Low	***
16	2015	Park, K.H. et al.	There is no difference in conservative treatment and surgical instrumentation. A prolonged antibiotic treatment > 6 weeks reduces the risk of recurrence.	153	retrospective study	IV	Low	**
17	2014	Shiban, E. et al.	The results of our retrospective study show that surgical treatment of spondylodiscitis with a staged surgical approach (if needed) and a short1-2-week period of intravenous antibiotics followed by 3 months of oral antibiotics is appropriate for most patients in whom conservative treatment has failed or is not advisable.The choice of the used material has no impact on the healing rate.	113	retrospective study	IV	Low	**
18	2013	Özkan, N. et al.	PMMA is an feasible alternative for bone grafts or cages in the treatment of cervical vertebral osteomyelitis.	21	retrospective study	IV	Low	*
19	2010	Hempelmann, R.G. et al.	The morbidity and mortality suggest that this surgical treatment is not the therapy of first choice in high-risk septic patients,but may be considered in patients when conservative management has failed.	18	retrospective study	IV	Very low	
20	2009	Sobottke, R. et al.	If surgery is indicated the operative risks should be borne in mind, but advanced age should not be the crucial factor in decision-making.The quality of life is equal in both groups (operative vs. conservative)	32	retrospective study			
21	2021	Lee, J.H. et al.	Clinical Outcomes in Older Patients Aged over 75 Years Who Underwent Early Surgical Treatment for Pyogenic Vertebral Osteomyelitis	439	retrospective study	IV	Low	***
22	2021	Lackermair, S. et al.	Distribution of Underlying Causative Organisms, Patient Age, and Survival in Spontaneous spondylodiscitis with Special Focus on Elderly Patients	51	retrospective study	IV	Very low	
23	2020	Alas, H. et al	Comparative outcomes of operative relative to medical management of spondylodiscitis accounting for frailty status at presentation	116	retrospective study	IV	Low	**
24	2018	Shah, K. et al	Role of Frailty Scoring in the Assessment of Perioperative Mortality in Surgical Management of Tuberculous Spondylodiscitis in the Elderly	26	retrospective study	IV	Very low	*
25	2018	Dubost, J.J. et al	Primary infectious spondylodiscitis in 51 patients over 75 years old: A comparative study	152	retrospective study	IV	Low	**
26	2017	Courjon, J. et al	Pyogenic vertebral osteomyelitis of the elderly: Characteristics and outcomes	351	post-hoc analysis	III	moderate	***
27	2018	Aguilar-Company, J. et al	Native vertebral osteomyelitis in aged patients: distinctive features. An observational cohort study	247	retrospective study	IV	Low	**
28	2017	Shetty, A.P. et al	Tubercular spondylodiscitis in elderly is a more severe disease: a report of 66 consecutive patients	66	retrospective study	IV	low	*
29	2017	Amadoru, S. et al	Spinal infections in older people: an analysis of demographics, presenting features, microbiology and outcomes	53	retrospective study	IV	very low	*
30	2016	Kothari, M.K. et al	Surgical Management in Elderly Patients with Tuberculous Spondylodiscitis: Ten Year Mortality Audit Study	20	retrospective study	IV	very low	
31	2016	Kothari, M.K. et al	Short to Mid-Term Term Surgical Outcome Study with Posterior Only Approach on Tuberculous Spondylodiscitis in an Elderly Population	16	retrospective study	IV	very low	*

### Diagnosis

**Figure 2.**
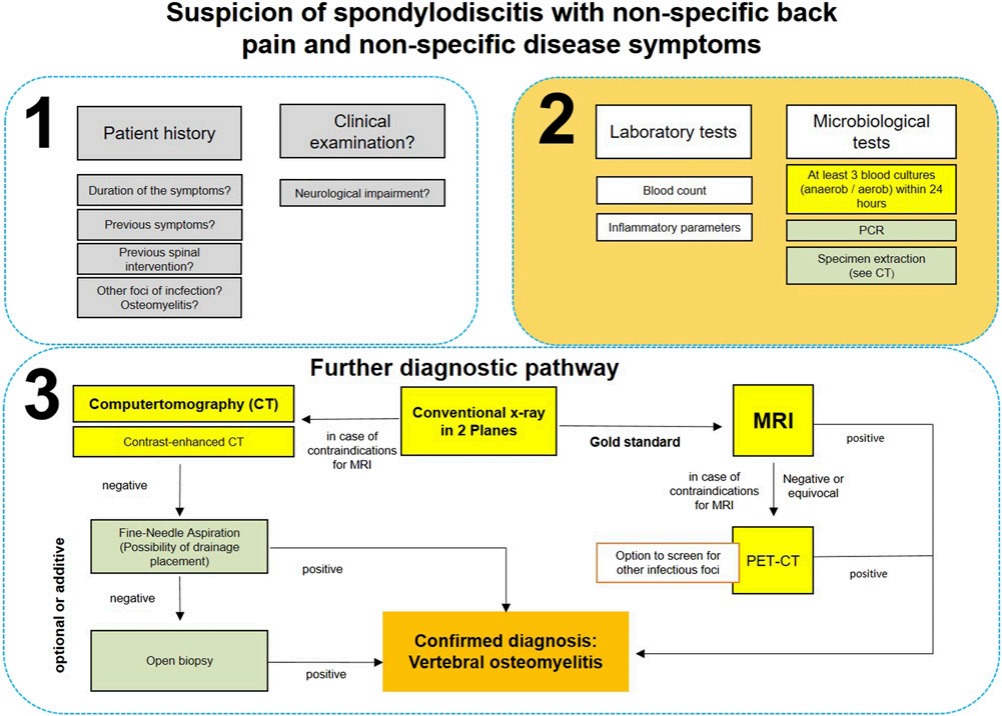
Diagnostic algorithm.

The search criteria used in this review showed only very few references for specific diagnostics especially in elderly patients (>65 years). Considering the current guidelines, the diagnostic workflow is identical in all age groups (>18 years). Therefore, the authors also implemented essential components from the guidelines of the USA 2015 (IDSA) and Germany 2020^12,13^ and present a diagnostic algorithm for suspected spondylodiscitis (Figure 2).

### Anamnesis and Laboratory Findings

In addition to radiological findings, the path to diagnosis is based on clinical, laboratory and microbiological results. It is not uncommon for this to cause a delay of 2-12 weeks between diagnosis and treatment initiation.^10^ The aim is therefore to reduce the time until the start of therapy by means of adequate diagnostics. Beside an extended clinical examination, a complete medical history based on the known red flags is essential (Table 2).^14^

**Table 2.** Anamnestic red flags for vertebral osteomyelitis.

B symptoms	Severe nocturnal pain
Past bacterial infection	Previous spinal infiltration treatment
Intravenous drug abuse	Immunosuppression
Underlying malignancy	Long-term stay abroad
Origin of the patient from a country with high incidence of tuberculosis	Haemodialysis

In all patients with existing back pain and suspected infectious origin, laboratory diagnostics are the basis for further diagnostics. A specific parameter proving the diagnosis of vertebral osteomyelitis does not exist. Laboratory tests includes leukocyte and C-reactive protein (CRP) counts. In literature, the blood sedimentation rate (ESR) is often mentioned as a parameter that is easy to determine, but it is non-specific, especially in the elderly.^15,16^ Amadoru et al showed that older patients with spinal infections present later, with higher markers of inflammation and fewer typical symptoms and signs of infection, leading to poorer outcomes.^17^ Leukocytosis is not necessarily present, whereas increased CRP is seen in 90%–98% of the cases.^10,18^ In case of clinical suspicion of a vertebral osteomyelitis, at least three blood culture pairs (aerobic/anaerobic) within 24 hours are obtained. The pathogen can be identified in 25%–59% of blood cultures, whereas a pathogen detection rate of as much as 70% is described in patients not previously treated with antibiotics.^10,19^

### Imaging Diagnostics and Findings

With increasing age, the likelihood of the presence of coexisting spinal pathologies also increases. Thus, the ability to distinguish clear radiographic signs of spondylodiscitis from other disease-related changes such as degenerative and inflammatory diseases and neoplasms of the spine becomes even more difficult.

Plain radiographs of the relevant spinal segment are the first-line imaging study in patients with unclear spinal symptoms. A negative x-ray does not rule out vertebral osteomyelitis but is nevertheless important to assess disease progression. Erosions and changes on the end plates of the corresponding segment being visible after weeks or months, depending on the pathogen virulence, the patient’s immune status or the clinical course (acute/chronic). However, there is no evidence in the literature showing destructive inflammatory processes to be more progressive or different in elderly patients than in young patients.^20^

Magnetic resonance imaging (MRI) is due to its very high sensitivity and specificity the golden standard in imaging studies to detect vertebral osteomyelitis, whereby adding a contrast agent also enables a distinction to be made between findings suspicious for vertebral osteomyelitis, degeneration (Modic type I), or neoplasia.^21,22^ With the help of MRI, both the extent of infection and possible skip lesions at other spinal levels can be shown. However, MRI often cannot be used in elderly patients with pacemakers or other electronic devices.

The computed tomography (CT) is primarily used for preoperative planning in order to be able to better evaluate the osseous situation, especially in osteoporotic bone. Depending on the system used, the preoperative performance of a thin-slice spiral CT can even be a prerequisite for the use of intraoperative navigation.^23^ In patients with MRI contraindications (non-MRI compatible pacemakers, other patient-specific factors), CT is the alternative diagnostic tool. By the use of CT, fine-needle biopsy or abscess drain placement is simplified.^24^

Fluorine-18 fluorodeoxyglucose positron emission tomography/CT (18F-FDG-PET- CT) is a well-known technique in oncology and infectious diseases and seems to play an increasingly important role in the diagnosis of vertebral osteomyelitis when MRI and CT are inconclusive or even in the first two weeks following disease onset.^25^ It represents a good alternative particularly in the case of contraindications to contrast-enhanced MRI/CT (eg, kidney failure), especially in the elderly. However, PET-CT’s lack of specificity to differentiate between neoplasia, spondylodiscitis, and post-traumatic bone marrow edema represents a drawback.^26,27^ Thus, its combination with MRI is recommended. Apart from the high costs, the use of PET/CT remains an individual decision, even if it makes sense to shorten the time to diagnosis, especially in geriatric patients. Diabetes mellitus is a typical disease of old age and must be taken into account. PET-CT examinations in diabetic patients with a sugar level of more than 150 mg/dl influence the quality of the results, and in severe cases the PET-CT cannot be performed at all.

## Material Acquisition: Puncture, Biopsy, Open Procedure

Material acquisition is the base for histopathological investigation and the start of potential antibiotic therapy. It can be obtained by CT-guided fine-needle biopsy or surgically removed. Pathogen detection is 19%–30% when using CT-guided fine-needle biopsy due to the small amount of tissue available, whereas detection can be achieved in 41% using histopathological methods [16]. A combination of local sampling and blood cultures therefore maximizes the likelihood of pathogen identification. According to recent systematic reviews, a pathogen can only be isolated in 33-60% of cases in geriatric patients.^28,29^ In addition to adequate abscess drainage, the diagnostic value of CT-guided fine-needle biopsy must certainly be questioned. Yang et al also suggest that percutaneous endoscopic discectomy and drainage yield higher bacterial recovery rates than CT-guided spinal biopsy.^30^ In any case, open sampling for histopathological and microbiological examination is more reliable, so that positive pathogen detection is possible in up to 68-93% of the cases.^31^

In the case of negative cultural results from the first biopsy and further clinical suspicion of spondylodiscitis, extended molecular biological tests (polymerase chain reaction) can be used to further identify the pathogen, especially in patients who have already been treated with antibiotics. Species-specific PCR (eg for *S. aureus* and *Mycobacterium tuberculosis*) can further increase sensitivity.^3,32^ However, the importance of nucleic acid-based detection methods (NAAT) in the diagnosis of spondylodiscitis has not yet been conclusively clarified. Individual studies provided indications of an increase in the sensitivity of pathogen detection, especially in connection with pathogens that are difficult to cultivate, when conventional culture-based methods were combined with NAAT.^32,47^ Choi et al were able to detect a total of 53.3% cases of spondylodiscitis using species-specific PCR, while only 28.9% could be detected using 16S rRNA-PCR.^32^ This was also shown by a large retrospective study in which 275 of 427 diagnoses (62.9%) could only be recorded by a species-specific PCR and not by a 16S rRNA-PCR.^48^ In contrast to the culture results, however, no statement can be made with the PCR on the antibiotic sensitivity of the pathogen. For the reasons mentioned above, NAAT is currently not part of routine diagnostics and is therefore reserved for rare diagnostic-scientific questions. (Figure 2)

## Non-Operative Treatment

Non-operative treatment is mainly based on pathogen-specific antimicrobial treatment. Some authors recommend spinal bracing, but evidence therefore is missing.

Three studies compared younger and elderly patients.^17,33,34^ Older patients seem to suffer more often from concomitant endocarditis, neoplasms, and chronic inflammatory disease.^33,34^ Regarding isolated pathogens, a decrease of *Staphylococcus aureus* and increase of Gram-negative pathogens was observed^17,33-35^ and a higher rate of streptococci^34^ and enterococci.^35^ Kim performed a retrospective study including 586 patients analyzing the pathogen distribution. Although *Staphylococcus aureus* was the most common pathogen it was more common in patients <60 years (53.7%; 60-75 years: 43.4%); >75 years: 32.5%) while Gram-negative bacteria were more common in older patients (≥60 years: 30.9%; <60 years: 14.7%; 60-75 years: 22%) as enterococci (≥60 years: 6,5%; <60 years: 0,7%; 60-75 years: 4%).^35^

In the elderly a higher mortality, treatment failure, rate of adverse events, longer hospital length of stay and lower quality of life was determined.^17,18,33,34,36^ Hutchinson reported that *Staphylococcus aureus* and *Streptococcus species* were the most common isolated pathogens. Intravenous therapy was performed for 36 days and in total 79.5 days. Patients had a mortality of 27%.^18^

Five studies compared conservative and surgical treatment.^37-41^ Conservatively patients were older, suffered from more comorbidities, and displayed a higher failure rate with increased 30-day mortality.^37-39^ Noteworthy, 90-day mortality and 1-year mortality were not significantly different.^39,41^ Furthermore, older patients had longer durations for their length of hospital stay, time to CRP normalization and intravenous antibiotic treatment but initially less high laboratory parameters and less common presence of abscesses.^37,39,41^ Quality of life and pain intensity did not differ between conservatively and surgically treated patients.^38,40^ Despite *Staphylococcus aureus* is still the most frequent pathogen in the elderly the rate of Gram-negative pathogen related spondylodiscitis seems to increase and should be considered especially in empirical antimicrobial therapy.^33,35^ Due to the high failure and 30-day mortality rate in the elderly as well as comorbidities conservative treatment should be considered critically especially regarding associated infections and their therapy.^33,39^ Patients with spondylodiscitis and epidural abscess formation display a high failure rate and should be treated surgically.^37^ No study examined if a longer treatment duration might be indicated for older patients to improve the outcome.

## Operative Treatment

If conservative treatment fails, surgical options must also be considered in geriatric patients.^42^ However, especially in this collective, surgical measures have their own morbidity and mortality risks.^38,42^ The complication rate in the surgically treated geriatric group of Sobottke et al was about twice as high compared to the conservative group.^43,44^

With regard to the surgical treatment strategy, the same paradigm applies to geriatric patients as to younger ones. The work of Kothari et al shows good short and medium-term postoperative results through a pure posterior treatment with regard to correction of the sagittal deformity and improvement of preoperative neurological deficits.^43^ In particular, the use of a pedicle screw/rod system was the best way to reconstruct the sagittal profile. Non-unions or recurrences did not occur in the collective.^43^ The results of Dreimann et al, who treated patients with thoracic spondylodiscitis via a purely dorsal approach using 360-degree stabilization and correction of the kyphosis, are comparable.^45^ Neither access-related nor pulmonary complications were observed. There was also no recurrence of the infection. The VAS was reduced from 8.8 preoperatively to 3.2, the sagittal profile could be significantly improved with a reduction of the thoracic kyphosis from 20° preoperatively to 7° after the operation. The results of Hempelmann et al also showed good clinical results in their geriatric patient collective (median age 72 years) after posterior stabilization and interbody fusion with iliac crest bone graft.^42^

However, pure decompression has also proven to be a sufficient treatment option in selected cases. Thus, Park et al compared collectives with debridement alone (median age 65 years) and debridement and instrumentation (median age 67 years).^46^ Clinical outcomes were comparable between groups, including the rate of infection-related deaths (2.1% vs 0%; P = .52), primary failure (1.1% vs 5.1%; P = .30) and frequency of recurrence (4.8% vs 6.8%; P = .72). Nevertheless, debridement alone should only be reserved for isolated epidural abscesses. In all other cases, additive stabilization makes sense and is necessary. Increased recurrence rates are not to be expected with sufficient antibiotic therapy.^46^

**Figure 3.**
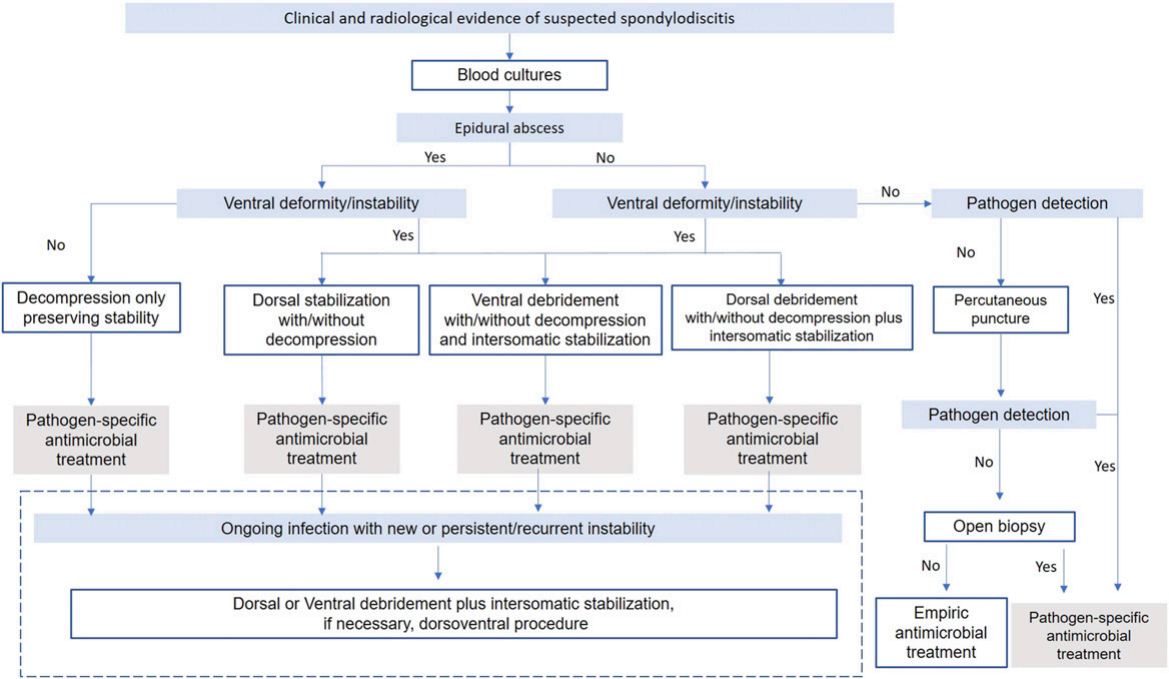
Treatment algorithm.

In summary, a dorsal approach in treatment of spondylodiscitis in the area of the lumbar and thoracic spine has proven itself in geriatric patients comparable to younger ones.^42,44,45^ However, as far as the ventral extent of destruction, the surgeon’s experience and the patient’s conditions allows, the operation should be expanded to include a one-stage ventral treatment, especially concerning spondylodiscitis in the cervical spine. The argument for a one- or two-stage ventro-dorsal surgical procedure is based on the theory that both stabilization and direct focus rehabilitation are possible (Figure3).

### Limitation

This is the first systematic literature review of the outcome and treatment of spondylodiscitis in elderly patients >65 years of age. The cutoff from 65 years of age may certainly create a bias. To provide an evidence-based assessment of the literature on this topic, this review was conducted in accordance with the PRISMA statement and used the GRADE approach. We limited our search to articles published worldwide in English and German language. This may have led to selection bias in our literature review. In addition, life expectancies vary across countries, so this may also be a source of bias. However, we set as the focus of our search to review the outcomes of both conservative and surgical treatment of pyogenic spondylodiscitis. Therefore, this review contains a very heterogeneous study population. Furthermore, this heterogeneity is reflected in the individual study populations of the included articles.

In terms of risk of bias, each included study was graded using the Newcastle-Ottawa scale (Table 1). The small sample sizes of most studies as well as the low level of evidence and missing prospective studies limit these recommendations.

## Conclusion

The incidence of spondylodiscitis increases by age with a peak at 75 years or older. The 1-year mortality is up to 20%. In conventional radiographs of the affected spinal segment, radiological signs of destruction are only seen in advanced stages of disease. If there is a medical history and clinical suspicion of spondylodiscitis, MRI with contrast agent of the of the spine is the Gold Standard for diagnostics. Geriatric patients show initially lower inflammatory parameters, have a longer time of hospitality, and take longer until inflammatory parameters normalize. The most frequent pathogen is Staphylococcus aureus. With increasing age, the rate of Gram-negative pathogens increases. Due to the high failure and mortality rate in the elderly patients, conservative treatment should be considered critically especially regarding associated infections and their therapy.

The main therapeutic goal in the treatment of spondylodiscitis is to achieve a recovery to improve the patient’s quality of life through mobility and pain relief. For this purpose, first the causative pathogens must be identified and thus a specific antibiotic therapy must be initiated.

Conservative and operative treatment must take the concomitant diseases into account. Patients with spinal instability, immobilizing pain, epidural abscess, and newly emerged neurological deficits should be considered for operative treatment. However, in all surgical procedures the presence of an osteoporotic geriatric bone structure should be expected.
